# HPV prevalence and HPV-related dysplasia in elderly women

**DOI:** 10.1371/journal.pone.0189300

**Published:** 2018-01-10

**Authors:** Ruth S. Hermansson, Matts Olovsson, Emelie Hoxell, Annika K. Lindström

**Affiliations:** 1 Department of Women's and Children’s Health, Uppsala University, Uppsala, Sweden; 2 Department of Oncology, Faculty of Medicine and Health, Örebro University, Örebro, Sweden; 3 Center for Clinical Research, Dalarna, Uppsala University, Uppsala, Sweden; 4 Clinical Research Center, Faculty of Medicine and Health, Örebro University, Örebro, Sweden; Georgetown University, UNITED STATES

## Abstract

**Introduction:**

In Sweden, where screening ends at the age of 60, about 30% of the cervical cancer cases occur in women older than 60. The aim of the present study was to investigate the prevalence of HPV and cervical dysplasia in women of 60 years and above.

**Patients and methods:**

From September 2013 until June 2015, 1051 women aged 60–89 years (mean 68 years) were sampled for an HPV test when attending an outpatient gynecology clinic. Women with positive results had a second HPV test and liquid based cytology (LBC), after 3.5 months on average. Those with a positive second HPV test were examined by colposcopy, and biopsy and a sample for LBC was obtained.

**Results:**

The prevalence of HPV was 4.1%, (95%CI 3.0–5.5, n = 43) at the first test, and at the second test 2.6% remained positive (95%CI 1.7–3.8, n = 27). The majority of women positive in both HPV tests, had dysplasia in histology, 81.5% (22/27) (4 CIN 2–0.4%, 18 CIN 1–1.7%). HPV-related dysplasia was found in 2.1%, (95%CI 1.3–3.2, n = 22) of the 1051 women. Four of the 22 women with positive HPV tests also had abnormal cytology, one ASCUS and three CIN 1. No cancer or glandular dysplasia was detected.

**Conclusion:**

A significant proportion of elderly women were found to have a persistent cervical HPV infection. Among them there was a high prevalence of CIN diagnosed by histology. The HPV test showed high sensitivity and specificity in detecting CIN in elderly women, while cytology showed extremely low sensitivity.

## Introduction

There is a lack of knowledge concerning the prevalence of infection with oncogenic types of human papilloma virus (HPV), and cervical dysplasia in women of 60 years and older. Older women often ask for continued screening for cervical cancer (CC) after the organized screening is terminated. In Sweden, a combination of organized and opportunistic Pap smear screening has reduced the incidence of squamous cell cancer in cohorts most regularly screened, by around 70% [1]. The present screening program ends at the age of 60. The Board of Health and Welfare recommend screening with an HPV test for women of 30 to 64 years of age, to be implemented during 2017 [2]. At present around 500 new cases of CC are diagnosed in Sweden annually, and the mortality is about 170 per year [3,4]. The incidence of CC has decreased in the age groups included in the screening program. About 30% of the cases of CC occur in women older than 60 and the mortality rate is about 70% in this age group [3,5,6]. Cervical cancer in women above the age of 65 is usually discovered at advanced stages and the prognosis is poor [6]. Women previously treated for cervical intraepithelial neoplasia grade 3 (CIN 3), are at increased risk of developing and dying from cervical or vaginal cancer, compared with the general female population. The risk accelerates above 60 years of age, suggesting a need for lifelong surveillance of these women [7]. During the past century the average life expectancy in Sweden has risen from 55 to 84 years and many women over 65 are healthy, continue to work [8], and have an active sex life [9].

Persistent infection with oncogenic types of human papilloma virus (HPV) is the major cause of cervical cancer [10]. As HPV DNA tests are more sensitive than cytology to detect pre-malignant lesions on the cervix, this method is now preferred as a primary screening method [11,12]. Repeat testing for HPV can be used to increase the specificity in the screening for cervical cancer [13].

In post-menopausal women, due to hormonal changes, the transformation zone where precursor lesions develop, is situated in the cervical canal and is therefore not accessible for proper examination and sampling [14]. As a consequence Pap smear for conventional cytology or liquid based cytology (LBC), has a low sensitivity and diagnostic surveillance with colposcopy for biopsy has little value [15]. Several studies have shown that screening with cytology has a low sensitivity in post-menopausal women [16–19]. There is limited knowledge regarding women older than 60, concerning the prevalence of HPV and its association with dysplasia and we have not been able to identify any studies focusing on this age group [20]. The aim of the present study therefore was to investigate the prevalence of HPV and cervical dysplasia in women of 60 years and older.

## Methods

**Fig 1:**
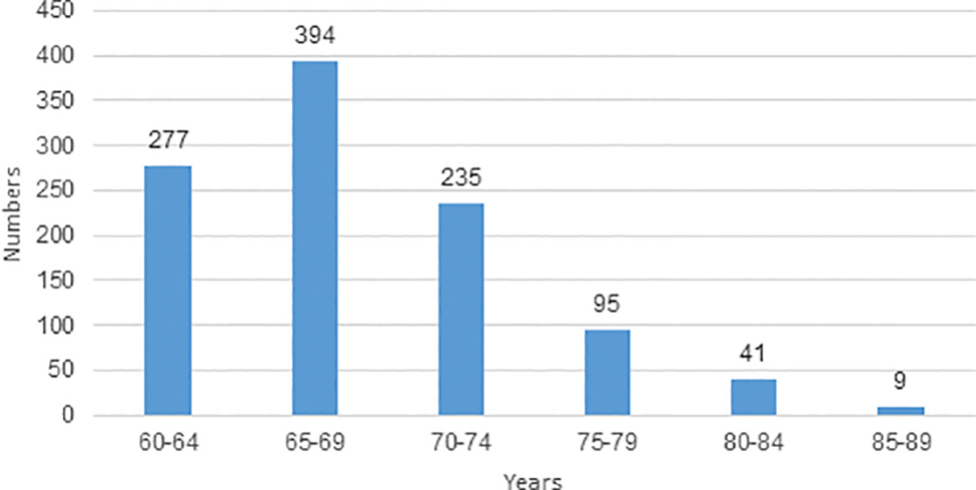
Age distribution of women sampled for the analysis of HPV (n = 1051).

This retrospective study is based on 1051 women aged 60–89 years (mean age 68 years), attending an outpatient gynecology clinic and having an HPV test as part of a gynecological examination (Fig 1). The study period was from September 2013 until June 2015. Women lacking the cervix, and those with a previous negative Pap smear within two years, were not included in the study. Participants having a positive first HPV test were re-examined after 3.5 months on average, including a second HPV test and a sample for LBC. Those who were HPV positive in the second test were referred to the outpatient clinic at the local hospital (Mora Hospital) for colposcopy, biopsy and second LBC. Women who tested negative in the second test had a third HPV test after one year. All HPV tests, colposcopies and LBCs were performed by one of the authors (AKL). The majority of the colposcopies with biopsies, cervical abrasions and conizations, were performed at the local hospital in Mora by one of the authors (RSH).

All LBC specimens were screened by cyto-technicians and those considered as abnormal were reviewed by a surgical pathologist. For cervical cytology The Thin Prep Pap Test was used. The cervical smear was collected with a plastic spatula and a cyto-brush. LBC specimens were placed in PreserveCyt solution and processed in the Thin Prep 5000-processor, HologicCytyc Corporation, Boxborough, Mass.) [21]. Specialists in surgical pathology examined the cervical biopsy samples and cones for histologic diagnosis. One senior pathologist re-evaluated all LBCs, cervical biopsies and cones, focusing on glandular atypia and adenocarcinoma.

The HPV test was performed using a multiplex real-time PCR assay (hpViR) as earlier described, which detects the following high-risk HPV types 16,18,31,33,35,39,45,51,52,56,58 and 59 (18 and 45 are detected together, and 33,52 and 58 as one group). A cervical sample was collected using a cytobrush. The sample was applied to a filter paper matrix, an indicating FTA elute card (Whatman, Inc., Clifton, New Yersey, rt no WB120411 [22,23]. The DNA was obtained from the FTA cards as described earlier [23]. The threshold for a positive HPV type was set to 10 copies per PCR [22].

The primary end point measurements were HPV infection and precursor lesions verified by cytology and histology. History of dysplasia in screening data back to 1986 was obtained for the HPV positive women.

The Regional Ethical Review Board, Uppsala, Sweden approved the study (Dnr 2015/136). Data was accessed anonymously. This retrospective study on clinical data did not require patient consent according to the Ethical Review Board. The HPV positive women have given written informed consent for historical data as approved by the Regional Ethical Review Board, Uppsala, Sweden (Dnr 2016/441).

For statistical analysis the Statistical Package for Social Sciences (SPSS) version 22 for Windows and Excel were used. A p-value less than 0.05 was considered statistically significant. The age groups 60–64 years (n = 277), 65–69 years (n = 394), 70–74 years (n = 235), 75–79 years (n = 95), 80–84 years (n = 41) 85–89 years (n = 9) and 60–69 years (n = 671), 70–79 years (n = 330), 80–89 years (n = 50) were compared. For statistical significance testing between age groups the chi-squared test in SPSS was used. Confidence intervals (CI) of proportions (Fleiss) were calculated using Excel [24].

## Results

**Fig 2:**
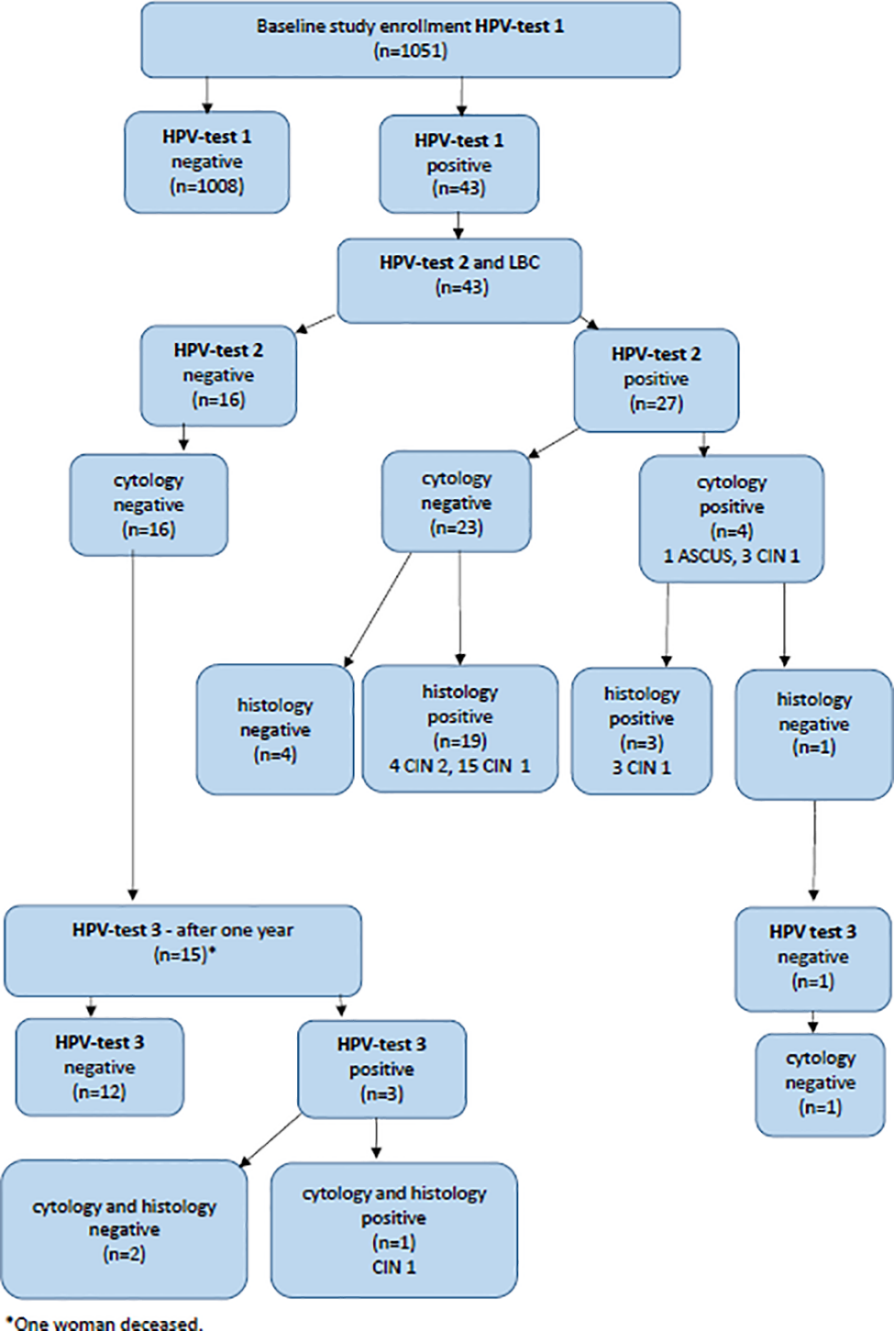
Flow chart showing study design and HPV and dysplasia occurrence (n = 1051).

All 1051 women offered testing, accepted an HPV test, and all samples were considered adequate and analyzed. The study design and HPV and dysplasia occurrence is shown in Fig 2 as a flowchart. All HPV positive women but one, were followed up for 22 to 43 months. Forty-three women (4.1%, 95% CI 3.0–5.5) were positive for HPV in their first test. In a second test, on average 3.5 months later, 27 women (2.6%, 95% CI 1.7–3.8) were still positive. All HPV positive women (n = 43) had a cervical smear for LBC collected at the same time as the second sample for HPV test was collected. Of the women with a positive second HPV test 81.5% (22/27) had dysplasia in histology, where four had CIN 2, and 18 CIN 1. HPV-related dysplasia, CIN 1–2, in the study population was thus 2.1% (95%CI 1.3–3.2) and CIN 2 alone was 0.4% (95%CI 0.1–1.0).

Of the 27 women that were positive for HPV in the second test, four had either atypical squamous cells of undetermined significance (ASC-US) or CIN 1 in cytology. One of them had benign histology and turned HPV and cytology negative after one year. Of the 22 women with dysplasia in histology, 19 (86.4%) had a normal cytology while 3 (13.6%) had dysplasia (1 ASC-US, 2 CIN I) also in the cytology. Women who were HPV negative in the second HPV test, 37.2% (16/43), all had a normal cytology and were scheduled for a follow up HPV test after one year.

In the third HPV test of 15 women after one year (one woman was deceased due to non-gynecological disease), 2 (13.3%) women were HPV positive with the same HPV type as in the first test. Cytology and histology showed CIN I in one woman and was normal in the other. There was no atypia of glandular cells, adenocarcinoma in situ or adenocarcinoma found. In screening history (from 1986 and onward) of the 43 women that had a positive first HPV-test 16 (37.2%) had a history of dysplasia (10 CIN 1, 2 CIN 2, 2 CIN 3, 2 others). Ten of these women (23.3%) had dysplasia before 60 years of age and 6 women when older than 60.

**Fig 3:**
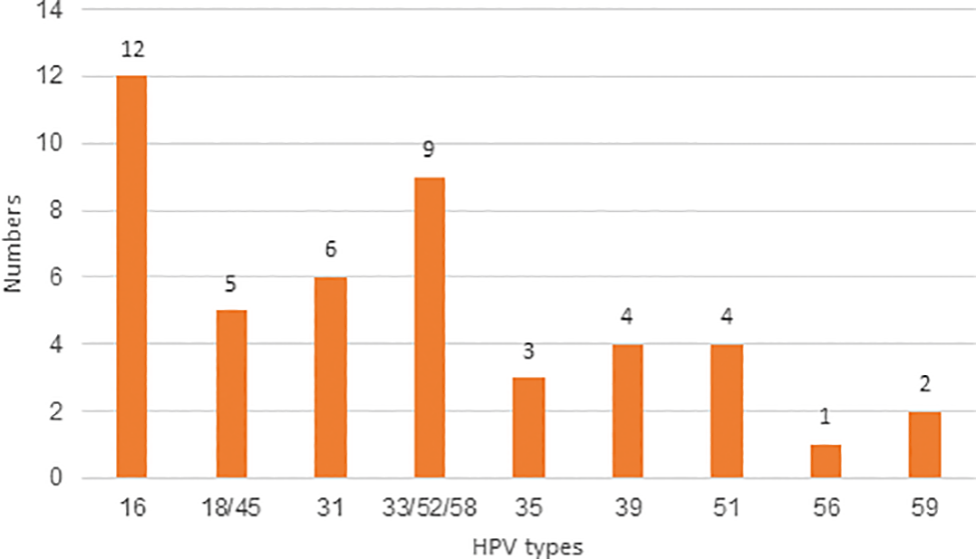
HPV types at first HPV test (n = 43, 3 women had multiple HPV infection).

**Fig 4:**
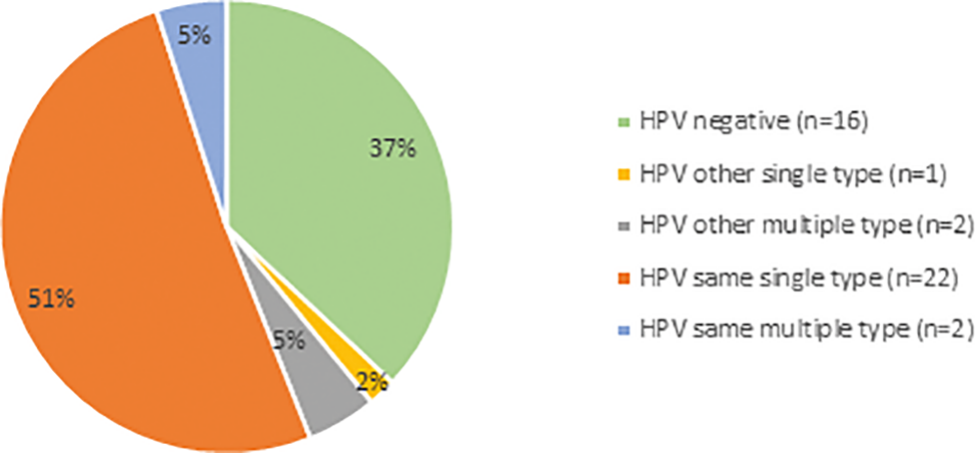
HPV types at second HPV test compared with the first HPV test (n = 43).

All HPV types tested for were found (Fig 3). Multiple infections were found in three women in the first test, and one of them was HPV negative in the second test. In the second test one of these women had a multiple infection with the same HPV types. Two other women had a single type infection in the first test but multiple infections in the second test. Of all 27 (62.8%) who tested positive in the second HPV test, 7.4% (n = 2) had shifted HPV type from the first to the second test (Fig 4).

At colposcopy of 26 of the 27 women who were also HPV positive in the second HPV test, the transformation zone (TZ) was not visible in 17 (65.4%), and partly visible in 9 (34.6%) of the cases. No one had a fully visible TZ. Vaginal lesions were found with colposcopy in three cases and biopsy for histological evaluation showed vaginal intraepithelial dysplasia VAIN I in all three cases. All three women with VAIN also had CIN I.

**Fig 5:**
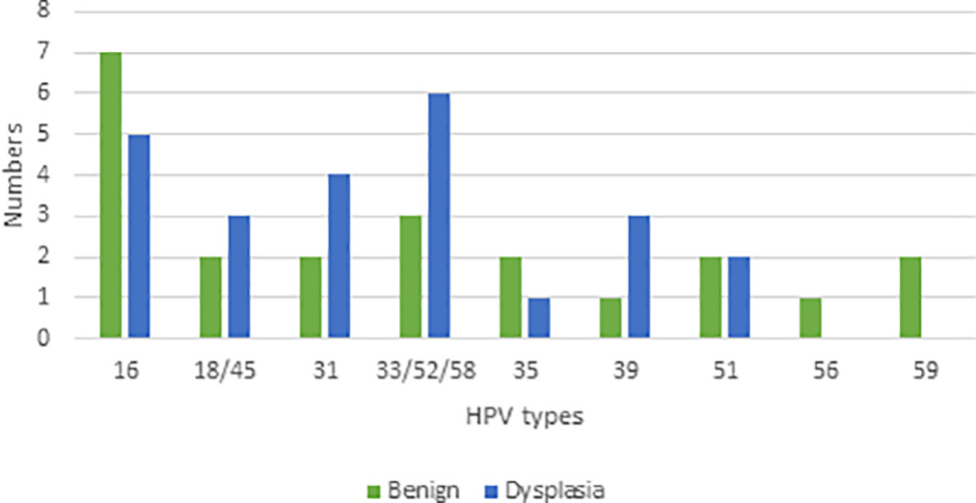
HPV types at first HPV test in women with benign histology vs. dysplasia (n = 43, where 3 women had multiple infection).

There were no differences in prevalence of HPV or dysplasia between age groups (5 years and 10 years), and there were no HPV positive women above the age of 80 (n = 50). The most prevalent HPV types were HPV 16 (27.9%) 12/43, followed by HPV 33/52/58 (20.9%) and HPV 31 (14.0%) (Fig 3). The distribution of HPV types in patients with dysplasia is shown in Fig 5. Conization was performed on 20 HPV-positive women, and of them 15 of 19 (78.9%) were HPV negative at follow up, on average 15 months (9–36 months) after surgery.

## Discussion

To our knowledge this is the first study on the prevalence of HPV and the association between HPV and dysplasia in elderly women. We found that the HPV prevalence was rather low in older women, but if they also tested positive in a second HPV test, the risk of cervical dysplasia was high. Since dysplasia was not detected by cytology in the vast majority of cases, LBC does not seem to be an appropriate method for screening women older than 60. In Sweden women are invited to the screening program until the age of 60, meaning that the current study focused on women who are no longer screened.

In the current study 4.1% of the women tested were HPV positive. As a comparison Ferenczy et al found that only 1% were HPV positive, out of 306 tested postmenopausal women aged 50–70 years [25]. The low prevalence could be due to a potential selection bias since these women had attended a Jewish hospital or clinics in private practice. Petignat and co-workers on the other hand found an HPV prevalence of 6.7% in women over 60, but the mean or median age is unfortunately not mentioned [26]. The reason why the different studies show such large differences in HPV prevalence, is probably due to different populations rather than different age groups or methods used to analyze for HPV. We believe that our data is reliable up to the age of 75, but not in women older than 75, since their number is rather small. A limitation of the current study is clearly the rather few women in the older age groups and the fact that it was carried out in one region of Sweden.

HPV clearance between test one and test two was as high as 37.2% after on average 3.5 months. This is surprisingly high since a study on women aged 30–50 years showed 29% clearance after on average 4.4 months (personal communication). In another study on women aged 30–65 years the clearance was 41% with 2.7 months between test one and test two [13]. It has previously been shown that an HPV infection persists longer in women older than 30 years compared with younger women [27,28]. A general belief is that the clearance from an HPV infection is a quicker process in pre- than postmenopausal women, but this might not however be true. The reason why 13% were positive with the same HPV type in the third HPV test as in the first HPV test, one year after the negative second test, is most probably explained by being a latent infection with no, or limited viral replication, or that the result from the HPV test was below the threshold for a positivity when the second HPV test was done. It has been shown that about 1 in 10 women who appear to have cleared their HPV infection may be latently infected [29].

We found poor correlation between LBC and histology, with only three with a positive cytology out of the 22 with dysplasia in histology, i.e. 13%. Cytology is thus not an appropriate method for the detection of cervical dysplasia in this age group. We also found that almost half of the women had a nonvisible TZ and none had a fully visible TZ. Colposcopy thus gave poor guidance for taking biopsy, as the transformation zone was not fully visible. We suggest this constitutes a potential sampling error for both cytology and histology due to an inability to visualize and sample from the TZ, which is the origin for precancerous lesions [14,30,31].

Biopsies lacking the TZ are not reliable and thus diagnostic conization should be offered to women with negative biopsies. In our experience there are additional difficulties in diagnostics in older women, due to vaginal atrophy and sometimes also vulvar disease such as lichen sclerosis.

Despite accounting for known risk factors, Charlton et al. were unable to predict individual patients' probability for progression of low grade squamous epithelial dysplasia (LSIL), to high grade dysplasia (HSIL) and adenocarcinoma in situ (AIS), on the basis of available data in women 16–66 years (mean 30.5 years) [32]. In younger women CIN2+ is the treatment threshold but in menopausal women this might not be optimal. Treating CIN 1 in older women might lead to fewer controls and the potential harm is less, since there is no risk of obstetrical complications due to a shorter cervix. In this study there was a high proportion of HPV negativity after conization at the short term follow up. Yet the optimal strategy, in terms of preventing cervical cancer in postmenopausal women, will depend on the degree to which precancerous changes progress to cancer or spontaneously regress, and this is not studied in elderly woman. Unfortunately, there is still no test to determine which precancerous lesions progress to cancer or spontaneously regress.

There is a need for future studies to form a basis for a screening algorithm adapted for postmenopausal women. In our experience Swedish women exit the screening program sometimes with the misleading information that they are not at risk for cervical cancer. Recently the Swedish Board of Health and Welfare has recommended a screening program based on HPV testing, which is extended until the age of 64 [2]. Our clinical experience is that many older women ask for cervical cancer screening. The knowledge that about 30% of the cases of CC occur in women older than 60, and that the mortality rate is as high as about 70% in this age group, should encourage an extension of the screening programs to also include older women.

## Conclusions

We hope that our results will serve as motivation to conduct studies focusing on older women also, in order to effectively reduce the prevalence of cervical cancer in this age group. Moreover it is clear that persistent cervical HPV infection is common in this age group and that the vast majority have cervical dysplasia. It is also clear that cytologically-based screening is not meaningful in this age group due to the low sensitivity of this method. The next step would be to introduce repeat HPV testing as a screening method and to find algorithms for sampling strategies such as self-sampling, test intervals and treatment options.

## Supporting information

S1 Dataset Supporting file. Raw data PLOS ONE.
(XLSX)

## References

[1] Bergstrom R, Sparen P, Adami HO (1999) Trends in cancer of the cervix uteri in Sweden following cytological screening. Br J Cancer 81: 159–166. doi: 10.1038/sj.bjc.6690666 10487628 10.1038/sj.bjc.6690666 PMC2374360

[2] Socialstyrelsen (2015) Screening för livmoderhalscancer Rekommendation och behandlingsunderlag Sweden: Socialstyrelsen;2015 1–38. www.socialstyrelsen.se.juni2015.

[3] The Board of Health and Welfare CfE (2015.) Cancer incidence in Sweden 2014. www.sos.se.

[4] The Board of Health and Welfare CfE (2015) Causes of Death 2013. www.sos.se. pp. 277.

[5] Socialstyrelsen (2011) Dödsorsaker 2009.

[6] Darlin L, Borgfeldt C, Widen E, Kannisto P (2014) Elderly women above screening age diagnosed with cervical cancer have a worse prognosis. Anticancer Res 34: 5147–5151. 25202106

[7] Strander B, Hallgren J, Sparen P (2014) Effect of ageing on cervical or vaginal cancer in Swedish women previously treated for cervical intraepithelial neoplasia grade 3: population based cohort study of long term incidence and mortality. BMJ 348: f7361 doi: 10.1136/bmj.f7361 24423603 10.1136/bmj.f7361 PMC3898577

[8] Commission E. Health at a glance: Europe 2014. Brussels: http://ec.europa.eu/health/reports/european/health_glance_2014_en.htm [2015-03-23]. pp. web page.

[9] Beckman N, Waern M, Gustafson D, Skoog I (2008) Secular trends in self reported sexual activity and satisfaction in Swedish 70 year olds: cross sectional survey of four populations, 1971–2001. BMJ 337: a279 doi: 10.1136/bmj.a279 18614505 10.1136/bmj.a279 PMC2483873

[10] Walboomers JM, Jacobs MV, Manos MM, Bosch FX, Kummer JA, et al (1999) Human papillomavirus is a necessary cause of invasive cervical cancer worldwide. J Pathol 189: 12–19. doi: 10.1002/(SICI)1096-9896(199909)189:1<12::AID-PATH431>3.0.CO;2-F 10451482 10.1002/(SICI)1096-9896(199909)189:1<12::AID-PATH431>3.0.CO;2-F

[11] Meijer CJ, Berkhof J, Castle PE, Hesselink AT, Franco EL, et al (2009) Guidelines for human papillomavirus DNA test requirements for primary cervical cancer screening in women 30 years and older. Int J Cancer 124: 516–520. doi: 10.1002/ijc.24010 18973271 10.1002/ijc.24010 PMC2789446

[12] Ronco G, Dillner J, Elfstrom KM, Tunesi S, Snijders PJ, et al (2014) Efficacy of HPV-based screening for prevention of invasive cervical cancer: follow-up of four European randomised controlled trials. Lancet 383: 524–532. doi: 10.1016/S0140-6736(13)62218-7 24192252 10.1016/S0140-6736(13)62218-7

[13] Gyllensten U, Sanner K, Gustavsson I, Lindell M, Wikstrom I, et al (2011) Short-time repeat high-risk HPV testing by self-sampling for screening of cervical cancer. Br J Cancer 105: 694–697. doi: 10.1038/bjc.2011.277 21811250 10.1038/bjc.2011.277 PMC3188941

[14] Tranbaloc P (2008) [Natural history of precursor lesions of cervical cancer]. Gynecol Obstet Fertil 36: 650–655. doi: 10.1016/j.gyobfe.2008.03.016 18539071 10.1016/j.gyobfe.2008.03.016

[15] Gilani SM, Mazzara PF (2013) Cytohistologic correlation in premenopausal and postmenopausal women. Acta Cytol 57: 575–580. doi: 10.1159/000353769 24107547 10.1159/000353769

[16] Gyllensten U, Gustavsson I, Lindell M, Wilander E Primary high-risk HPV screening for cervical cancer in post-menopausal women. Gynecol Oncol 125: 343–345. doi: 10.1016/j.ygyno.2012.01.036 22293044 10.1016/j.ygyno.2012.01.036

[17] Gyllensten U, Lindell M, Gustafsson I, Wilander E HPV test shows low sensitivity of Pap screen in older women. Lancet Oncol 11: 509–510; author reply 510–501. doi: 10.1016/S1470-2045(10)70064-4 20522375 10.1016/S1470-2045(10)70064-4

[18] Gustafsson L, Sparen P, Gustafsson M, Pettersson B, Wilander E, et al (1995) Low efficiency of cytologic screening for cancer in situ of the cervix in older women. Int J Cancer 63: 804–809. 8847138 10.1002/ijc.2910630610

[19] Colgan TJ, Clarke A, Hakh N, Seidenfeld A (2002) Screening for cervical disease in mature women: strategies for improvement. Cancer 96: 195–203. doi: 10.1002/cncr.10723 12209660 10.1002/cncr.10723

[20] de Sanjose S, Diaz M, Castellsague X, Clifford G, Bruni L, et al (2007) Worldwide prevalence and genotype distribution of cervical human papillomavirus DNA in women with normal cytology: a meta-analysis. Lancet Infect Dis 7: 453–459. doi: 10.1016/S1473-3099(07)70158-5 17597569 10.1016/S1473-3099(07)70158-5

[21] Carpenter AB, Davey DD (1999) ThinPrep Pap Test: performance and biopsy follow-up in a university hospital. Cancer 87: 105–112. 10385440 10.1002/(sici)1097-0142(19990625)87:3<105::aid-cncr2>3.0.co;2-z

[22] Gustavsson I, Juko-Pecirep I, Backlund I, Wilander E, Gyllensten U (2009) Comparison between the Hybrid Capture 2 and the hpVIR real-time PCR for detection of human papillomavirus in women with ASCUS or low grade dysplasia. J Clin Virol 45: 85–89. doi: 10.1016/j.jcv.2009.04.012 19451022 10.1016/j.jcv.2009.04.012

[23] Gustavsson I, Lindell M, Wilander E, Strand A, Gyllensten U (2009) Use of FTA card for dry collection, transportation and storage of cervical cell specimen to detect high-risk HPV. J Clin Virol 46: 112–116. doi: 10.1016/j.jcv.2009.06.021 19628427 10.1016/j.jcv.2009.06.021

[24] Fleiss JL (1981) Statistical methods for rates and proportions.: John Wiley & Sons, Inc.

[25] Ferenczy A, Gelfand MM, Franco E, Mansour N (1997) Human papillomavirus infection in postmenopausal women with and without hormone therapy. Obstet Gynecol 90: 7–11. doi: 10.1016/S0029-7844(97)00217-2 9207803 10.1016/S0029-7844(97)00217-2

[26] Petignat P, Faltin D, Goffin F, Billieux MH, Stucki D, et al (2005) Age-related performance of human papillomavirus testing used as an adjunct to cytology for cervical carcinoma screening in a population with a low incidence of cervical carcinoma. Cancer 105: 126–132. doi: 10.1002/cncr.21060 15822123 10.1002/cncr.21060

[27] Rodriguez AC, Schiffman M, Herrero R, Hildesheim A, Bratti C, et al (2010) Longitudinal study of human papillomavirus persistence and cervical intraepithelial neoplasia grade 2/3: critical role of duration of infection. J Natl Cancer Inst 102: 315–324. doi: 10.1093/jnci/djq001 20157096 10.1093/jnci/djq001 PMC2831050

[28] Plummer M, Schiffman M, Castle PE, Maucort-Boulch D, Wheeler CM, et al (2007) A 2-year prospective study of human papillomavirus persistence among women with a cytological diagnosis of atypical squamous cells of undetermined significance or low-grade squamous intraepithelial lesion. J Infect Dis 195: 1582–1589. doi: 10.1086/516784 17471427 10.1086/516784

[29] Korostil IA, Regan DG (2014) The potential impact of HPV-16 reactivation on prevalence in older Australians. BMC Infect Dis 14: 312 doi: 10.1186/1471-2334-14-312 24906851 10.1186/1471-2334-14-312 PMC4061121

[30] Virtej P, Matei M, Badea M, Badea I, Popa O (1998) Cervical intraepithelial neoplasia and HPV infection. Eur J Gynaecol Oncol 19: 179–181. 9611062

[31] Aschkenazi-Steinberg SO, Spitzer BJ, Spitzer M (2005) The role treatment for cervical intraepithelial neoplasia plays in the disappearance of human papilloma virus. J Low Genit Tract Dis 9: 19–22. 15870517 10.1097/00128360-200501000-00005

[32] Charlton BM, Carwile JL, Michels KB, Feldman S (2013) A cervical abnormality risk prediction model: can we use clinical information to predict which patients with ASCUS/LSIL Pap tests will develop CIN 2/3 or AIS? J Low Genit Tract Dis 17: 242–247. doi: 10.1097/LGT.0b013e3182730fec 23486071 10.1097/LGT.0b013e3182730fec PMC3696437

